# Age-Dependent Fecal Bacterial Correlation to Inflammatory Bowel Disease for Newly Diagnosed Untreated Children

**DOI:** 10.1155/2013/302398

**Published:** 2013-04-18

**Authors:** Felix Chinweije Nwosu, Lill-Therse Thorkildsen, Ekaterina Avershina, Petr Ricanek, Gøri Perminow, Stephan Brackmann, Morten H. Vatn, Knut Rudi

**Affiliations:** ^1^Hedmark University College, Hamar, Norway; ^2^Department of Chemistry, Biotechnology and Food Science, Norwegian University for Life Sciences, Ås, Oslo, Norway; ^3^Department of Gastroenterology, Akershus University Hospital, Lørenskog, Norway; ^4^EpiGen Institute, Research Centre, Akershus University Hospital, Lørenskog, Norway; ^5^Pediatric Department, Oslo University Hospital, Ullevål, Oslo, Norway; ^6^EpiGen Institute, Akershus University Hospital, University of Oslo, Oslo, Norway; ^7^Medical Clinic, Oslo University Hospital, Rikshospitalet, Norway

## Abstract

The knowledge about correlation patterns between the fecal microbiota and inflammatory bowel diseases (IBD)—comprising the two subforms Crohn's disease (CD) and ulcerative colitis (UC)—for newly diagnosed untreated children is limited. To address this knowledge gap, a selection of faecal specimens (CD, *n* = 27 and UC, *n* = 16) and non-IBD controls (*n* = 30) children (age < 18 years) was analysed utilising bacterial small subunit (SSU) rRNA. We found, surprising age dependence for the fecal microbiota correlating to IBD. The most pronounced patterns were that *E. coli* was positively (*R* ^2^ = 0.16, *P* = 0.05) and Bacteroidetes, negatively (*R* ^2^ = 0.15, *P* = 0.05) correlated to age for CD patients. For UC, we found an apparent opposite age-related disease correlation for both *Bacteroides* and *Escherichia*. In addition, there was an overrepresentation of *Haemophilus* for the UC children. From our, results we propose a model where the aetiology of IBD is related to an on-going immunological development in children requiring different age-dependent bacterial stimuli. The impact of our findings could be a better age stratification for understanding and treating IBD in children.

## 1. Introduction

The human gut microbiota is represented by about 10^14^ microbes per individual, comprising more than 500 species. The human gut microbiota is dominated by the members of the phyla Bacteroidetes and Firmicutes [1, 2]. The interactive relationship, symbiosis or pathogenic, between the host and the gut bacteria is shaped by selective pressures within the host (genetic) and competitive modulation of resident microbial community, of which net effect may have implications on the health of the host [1, 3]. An imbalance in the composition of the commensals or beneficial (symbiotic) bacteria and pathogenic bacteria creates an abnormal host microbiota, which may lead to IBD or other diseased states [4–7].

From various IBD studies, etiologically implicated bacteria include *Faecalibacterium prausnitzii* exerting a positive impact, described as anti-inflammatory, while *Escherichia coli* and *Mycobacterium avium paratuberculosis* (MAP) have a negative impact as potential IBD infectious agents [5, 8–12]. These relations are mainly established from differences in gut bacterial composition in analysed fecal samples between observed IBD cases and healthy subjects [2].

The incidence of pediatric onset of IBD is increasing [13]. Compared to adults, there is an overrepresentation of CD over UC in pediatric patients, while UC is often more severe in children compared to adults [14]. There are several lines of evidence for strong disease-related correlation patterns with the gut microbiota in children [15]. However, the microbiota related to the onset of the disease is not yet well characterized [16]. 

The aim of the current work was to establish the correlation patterns between the composition of the dominant gut microbiota and IBD in newly diagnosed untreated children. Our strategy was to use a novel mixed 16S rRNA gene sequencing approach to describe the overall composition of the microbiota [17, 18] in combination with full-length 16S rRNA gene clone Sanger sequencing [19] to obtain strain/species level information. 

We present results showing age-related correlations between gut bacteria and IBD, in addition to average differences in the microbiota. We also present an explanation model for our observations.

## 2. Results and Discussion

### 2.1. Microbiota Composition in Study Population

**Figure 1:**
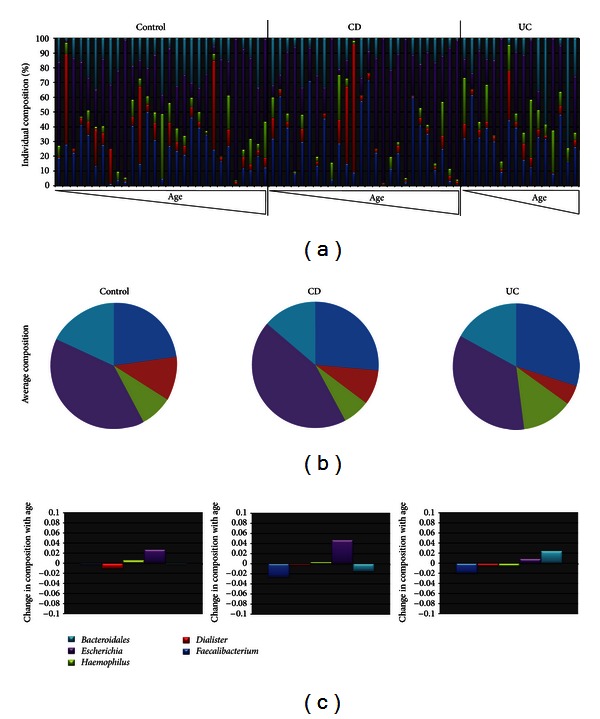
Composition of the gut microbiota as determined by MCR. (a) Individual distribution of the gut microbiota. (b) Average composition of the gut microbiota within the disease categories analysed. (c) Change of gut microbiota within disease categories with age.

Using principal component analysis (PCA), evolving factor analysis, and empirical evaluations, we found that our mixed sequences were composed of 6 main components (explaining the majority of the variance) representing the dominating phylogroups in the dataset. These were resolved by Multivariate Curve Resolution (MCR). Five of the components represented spectra that could be base called (Table 1), while the sixth component probably represented noise due to the short length and lack of match in the Ribosomal Database Project (RDP) database (not shown). The dominant taxa identified were *Faecalibacterium*, *Dialister, Haemophilus, Escherichia,* and* Bacteroides *for MCR components 1, 3, 4, 5 and 6, respectively. These taxa showed a relatively diverse distribution pattern among the children analysed (Figure 1, Supplementary Table 1, see Supplementary Material available online at http://dx.doi.org/10.1155/2013/302398). 

**Figure 2:**
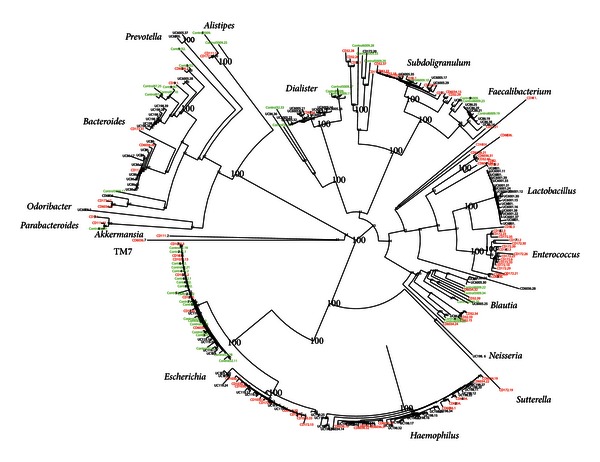
Neighbour joining phylogenetic tree for the nearly full-length sequences obtained in this work. The numbering at the nodes is the bootstrap support, with the 100% support values highlighted. For each sequence, the colour code indicates the patients diagnosis: green—control, red—CD, and black—UC.

Cloning and sequencing confirmed the main composition detected by MCR (Figure 2, Supplementary Table 2). In addition, we detected a range of taxa representing minor constituents in the clone data (Figure 2). 

### 2.2. IBD Correlation to the Dominant Microbiota

The main age-related trends in the data were both positive (*P* = 0.05) and negative (*P* = 0.05) age correlations for *Escherichia *and *Bacteroides*, respectively, for the CD children (Figure 1(c)). In addition, permutation testing revealed a significant increase in *Bacteroides *compared to the control and CD children (*P* = 0.01), although the slopes were not significantly different from zero. 

For the nonage-related patterns, *Escherichia* was underrepresented (*P* = 0.05) while *Haemophilus* was overrepresented (*P* = 0.01) in UC, as compared to CD and controls. For CD, on the other hand, we found an underrepresentation of *Haemophilus*  (*P* = 0.05) (Figure 1(b)). 

For the diversity analyses, we did not find any strong age-related trends, while we found that both Shannon's *H* and Simpson's *D* indexes were significantly lower for the CD subjects compared to the controls (0.32 versus 0.48 *P* = 0.05 and −0.46 versus −0.08 *P* = 0.04, resp.). For the UC children we did not find a significantly reduced diversity. 

The strain level correlations showed that there were two clades of* Escherichia*, one associated with diseased patients and another associated mainly with one of the control patients. Furthermore, it suggests that *Haemophilus *represents a very tight phylogroup, mainly associated with diseased patients. For CD, we detected a cluster of *Enterococcus*, while for UC a cluster of *Lactobacillus *was detected. These clusters, however, were only represented by single patients. 

### 2.3. Potential Causes for the IBD Bacteria Correlation

The apparent opposite age-related trend for *Bacteroides *and *Escherichia *between UC and CD may reflect the differences in underlying immunological disorders for these diseases. CD is a Th1 dominated immunological disease, and UC is a Th2 dominated immunological disease [16]. Therefore, a possible explanation could be that the immunological effects of *Bacteroides* at early age promote CD, while later it would protect. The development of the immune system in children is an on-going process potentially requiring different stimuli at different ages [20]. For *Bacteroides fragilis*—one of the most widely studied species within the Bacteroidetes—it has been shown that this bacterium can produce immunosuppressive polysaccharides with a potential therapeutic use for CD in adults [21]. This can explain the protective effect with age, while immune suppression at an earlier age may promote the disease. The positive age correlation for *Escherichia *and CD can be explained under the same model. It has been shown that exposure to *E. coli* early in life can promote the immune development in a Th1, as opposed to an allergenic Th2 direction [22–24]. In the adult or adolescent population, on the other hand, *E. coli* is associated with ileal CD [25]. Thus, it could be that the immune stimulatory effect at early age would be important for immune homeostasis, while at a later age similar stimulations would lead to a dysbiotic CD state.

We found that overrepresentation of *Haemophilus* in UC was interesting. Despite extensive screenings, no studies, have yet identified this bacterium as important in UC [15], while in our study this bacterium was significantly correlated to UC. The mucosal inflammation properties of several *Haemophilus* species and the requirement for blood factors for growth [26] may suggest that it could be important in the disease onset. However, further investigations are needed in order to rule out potential confounders such as water contamination, drug regimes, and collateral diseases. The number of individuals included in our study is also relatively low. 

We found the overall high level of *Escherichia *in the control group surprising. This is not expected in a healthy population [27]. However, since our control group was selected from children who were suspected to have IBD, but eventually diagnosed as non-IBD, these cannot be considered as representatives for the normal healthy population. The control samples probably represent a heterogeneous population of different forms of dysbioses since they are recruited based on IBD symptoms but eventually found to be non-IBD. These probably include inflammatory bowel syndrome (IBS) cases, with similarities in symptoms to IBD. Similarly, these subjects may have been IBD without full manifestation or on the border of disease development. 

In conclusion, the correlation patterns detected may reflect the underlying age-related disorders in IBD. 

## 3. Materials and Methods

### 3.1. Cohort

A total of 75 children samples (<18 years old) stored at −80°C were provided from diagnosed patients stool specimens. Samples were deposited from diagnosed, early inflammatory bowel disease (IBD) patients at Akershus University Hospital (Ahus), Oslo, Norway. These were the children from the Norwegian IBSEN II study for which we have stool samples. From these collections, 27 were diagnosed, Crohn's disease (CD), 16 diagnosed ulcerative colitis (UC), and 30 samples were from diagnosed non-IBD subjects (control). The criteria for diagnosis and detailed information about the subject are presented in Tables 2 and 3.

IBD diagnosis criteria for patient specimen included in the IBSEN II cohort were abdominal symptoms including diarrhea and/or blood in stool for more than 10 days and endoscopic or radiological examinations for signs of inflammation and histological signs of chronic inflammation. 

Subjects with pathogenic gut bacterial infection (except *Mycobacterium avium*), parasites, cysts, and eggs were excluded from this cohort. Similarly, comorbid patients with cancer, haematological or hepatological disorders, and significant cardiovascular, neurological, and respiratory conditions were not included in this study. In addition, other chronic inflammations were exempted from this study in both disease and control subjects.

### 3.2. DNA Purification and Quantification

 Between 180 and 220 mg of frozen stool (−80°C) was cut with a scalpel and transferred to each of 2 mL microcentrifuge tubes. These were mechanically and vigorously lysed with 1.6 mL of ASL buffer (Qiagen, Hilden, Germany) for 2 minutes at 30 Hz using magnetic beads (Qiagen, Hilden, Germany) on Qiagen TissueLyser (Qiagen, Hilden, Germany) and further lysed at 95°C for 5 minutes in a heating block. For the subsequent processing, we followed the recommendations of the producer (http://www.qiagen.com/MyQIAcube/). 

### 3.3. Polymerase Chain Reaction (PCR) Amplification of 16S rRNA

An approximately 1200 bp 16S rRNA gene region covering V3 to V9 was PCR amplified as previously described [28]. The reaction mix contained 1.25 U Hot FirePol (Solis Biodyne, Tartu, Estonia), 1 × B2 buffer (Solis Biodyne, Tartu, Estonia), 2.5 mM MgCl_2_, 200 *μ*M dNTP (Thermo Fisher Scientific, Surrey, UK) and 0.2 *μ*M each of forward and reverse primers to approximately 30 ng DNA template in final volume of 25 *μ*L. The PCR thermocycler was programmed for initial denaturation at 95°C for 15 minutes, with 30 cycles of denaturation at 95°C for 30 seconds, annealing for 30 seconds at 55°C, elongation for 1 minute and 20 seconds at 72°C, and at the end a final elongation for 7 minutes at 72°C.

### 3.4. Mixed Sequencing

The PCR product was firstly prepared for sequencing by treatment with 3U Exonuclease I (ExoI) and 8U, shrimp alkaline phosphatase. (USB Corp, OH, USA) at 37°C for 2 hours, and was inactivated at 80°C for 15 minutes. 

The ExoSAP treated PCR product was diluted 1/10, then 1 *μ*L was placed in each well and included with 0.32 *μ*M each of 5′-[C X30]CGTATTACCGCGGCTGCTGGCAC-3′ (U515FC30) primers, 1 × BigDye buffer and 1 *Μ*l, BigDye v1.1, incorporation reaction to a 10 *μ*L total volume. The PCR thermocycler was programmed at 25 cycles of denaturation at 96°C for 15 seconds, annealing at 50°C for 5 seconds and elongation at 60°C for 4 minutes. The sequencing reaction was cleaned using the XTerminator kit following the manufacturers' recommendations (Applied Biosystems). Sequences analysis was on the ABI Genetic Analyzer 3130xl sequencer with 36 cm capillary array containing polymer 7 (POP-7, Applied Biosystems). Injection time was set at 6 seconds at 90°C. The sequences generated were base called by the Sequence Scanner Software v1.0 (Applied Biosystems).

The mixed sequences were resolved using the Multivariate Curve Resolution (MCR) analysis to expose and recover the pure components in the spectral of sequences. MCR is a technique to resolve pure spectra. Firstly, an alignment of all of the mixed sequences spectra was generated and repeated for preprocessing and normalization taking only small portions of individual peaks in the spectra to avoid peak shifts due to differences in retention times. principal component analyses (PCA) and/or evolving factor analyses (EFA) determined the number of significant components explaining the most variations in the dataset. With the predetermined component number setting, MCR was run on the aligned spectra. MCR output is the information on the relative amount of each component in the individual sample/sequence in the data set and the base called spectra information on each component. All the analyses of sequence spectra were performed using MATLAB R2010a software (The MathWorks Inc., Natick, MA, USA), Statistical and Bioinformatics toolboxes for MATLAB. For EFA, PCA, and MCR analyses, PLS Toolbox v5.8 for MATLAB (Eigenvector Research Inc., USA) was used.

### 3.5. Cloning and Full-Length 16S rRNA Gene Sequencing

Using the MCR resolved sequence components, DNA pools were empirically selected for cloning from each component, 15 samples in total, corresponding to 3 for each component for each classification (UC, CD, and control). Amplicon cloning and DNA sequencing were done as previously described [18], sequencing both strands. Forward and reverse sequence reaction results were assembled, aligned, and trimmed for noise using the CLC Genomic Workbench Software (CLC bio A/S, Denmar). Aligned sequences were filtered for chimeric 16S rRNA sequences using the chimeric slayer algorithm in mothur (http://www.mothur.org/) prior to further analysis.

### 3.6. Ecological Diversity Analyses

We analysed ecological diversity using modified versions of Simpson's *D* and Shannon's *H*. In our case, we used the MCR components as species surrogate. The following formula was used for Simpson's *D*:

(1) $$\begin{array}{c} \text{Simpson's}\;D=1-\sum {\text{MCR}}_{i}. \end{array}$$

The modified Shannon's *H* was calculated using the following formula:

(2) Shannon's  H=∑MCRi×Log(MCRi),

where MCR_*i*_ is the score for the *i*th component.

## Supplementary Material

The Supplementary Material contains information about the microbiota composition in all the children analyzed (Supp. Table 1) and a classification of the cloned sequences (Suppl. Table 2).

## Figures and Tables

**Table 1:** Base-called MCR components.

Components (match)	Sequences
Comp1 (*Faecalibacterium*)	AGCGTGTCCGATTTACTGGGTGTAAGGGAGCGCTAGCGGAGAGCAAGTTCG GAGTGAAATCCATGGGCTCAACCCATGGAARTGCTTTCAAACCTTGMTTTT CTTTGYTAGTGCAAAGGTAAAGTCGGATRCCTGAGGTGGTACGGGTGGAAT GCGTAATATTYGGAGGAACACCATGGCAAGGCGGTCRTACTGGGCACCAAC TGACGRTGAGGCTCAA

Comp2 (designated noise)	AGCTAGTATCCGGATTCTARTGGGTGTAAAGGGCGTAGCGGTTATCTAAAGG GCTTTT

Comp3 (*Dialister*)	AGCGTTGTCCGGATTATTGGGCGTAAAGCGCGCGCAGGCGGCTTTCCRAAGTC CTCTCTTAAAAGTGCGGGGCTTAACCCCMGYTG GGGYATGYAACCTGGYAAYCCTGGAGTATCGGAYAGYAAAGMGAGAATTCCAT AGTGTAGCGGTYAAATGCGTAAGATTAGGAAG AACACCGGTGGCGAAGGSGACTTTCTGGACAAAACTGACGCTGAGGCGCGAAA

Comp4 (*Haemophilus*)	AGCGTTATTCGGAATAARTGGGCGTAAAGGGCACGCAGGCGGTGKCTTAAGTG AGGTGTGAAAGCGCCCGGGCTTAACCTGGGAATMGCATTTCATACTGGGGTGC CGTAAMTACTTTAGGGAGYGGTAYATATTCTCACGTMGTAGCGGTYAAWGTSC TTAAGTATGTGAAGYAATACCGAAGGCAGAAGSCARCCCTMGGAWTGTCACGT GACSRTCATGTGCAAA

Comp5 (*Escherichia*)	AGCGTTAATCGGAATTACTGGGCGTAAAGCGCACGCGGCGGTTTGTTAAGTCAG ATGTGAAATCCCCGGGCTCAACCTGGGAACTGCATCTGATACTGGCAAGCTTGA GTCTCGTAGAGGGGGGTAGAATTCCAGGTGTAGCGGTGAAATGCGTAGAGATC TGGAGGAATACCGGTGGCGAAGGCGGCCCCCTGGACGAAGACTGACGCTCAGG TGCGAAA

Comp6 (*Bacteroides*)	ACGTTATCCGGATTTATTGGGTTTAAAGGGAGCGTAGTGGARTTGTTAAGTCA TGTATGTGAAAGCTTTGCGGCTCAACCGTAAAATTGCATTTGAWACTGGAAGW CTTGAGTGCAGTAGAGGRAGAGGCGGAATTCCTGGTGTAGCGGTGAAATGCTAA TATCACGAAGAACATCCGATGTGCGAAGGCGGCTTAGCTGGACTGTAACTGACY RTGAMGCTCGAAA

**Table 2:** Demographic data.

Disease group	Number of patients	Average age	Median duration^1^	Male (%)^1^
CD	27	12.8	0.5	56
UC	16	11.3	0.4	47
Non-IBD	30	11.5	1.5	32

^1^Adapted from [13].

**Table 3:** Biochemical blood test levels and fecal calprotectin^1^.

Measurement	Unit	UC	CD	Non-IBD
ESR	mm/h	17	26	5
CRP	mg/L	7	22	7
Hemoglobin	g/dL	11	11.6	12.5
Hematocrit	Fractions	0.35	0.36	0.38
Platelet count	10^9^/L	373	368	268
Leukocytes	10^9^/L	7	8.5	6.8
Neutrophil granulocytes	10^9^/L	4.2	4.7	3.5
ALAT	U/L	18	16	24
Alkaline phosphatase	U/L	148	106	189
Albumin	g/L	42	37	40.5
Calprotectin	mg/kg	1181	1250	33

^1^Average values adapted from [13].
