# Supplementary material for: Age-Dependent Fecal Bacterial Correlation to Inflammatory Bowel Disease for Newly Diagnosed Untreated Children
Source: Gastroenterol Res Pract. 2013 Apr 18;2013:302398. doi: 10.1155/2013/302398 (PMC3652150; doi:10.1155/2013/302398)
Supplement: Supplementary file 1 — The Supplementary Material contains information about the microbiota composition in all the children analyzed (Supp. Table 1) and a classification of the cloned sequences (Suppl. Table 2). [file 302398.f1.docx]

**Suppl. Table 1. Individual distribution of the gut microbiota**

| **Patient** | **Age** | **Diag** | **av 1** | **av 3** | **av 4** | **av 5** | **av 6** | **std 1** | **std 3** | **std 4** | **std 5** | **std 6** |
| --- | --- | --- | --- | --- | --- | --- | --- | --- | --- | --- | --- | --- |
| 7.00 | 13.00 | CD | 1.19 | 0.07 | 0.01 | 0.24 | 0.15 | 0.03 | 0.01 | 0.01 | 0.02 | 0.04 |
| 28.00 | 12.00 | CD | 0.15 | 1.39 | 0.04 | 0.03 | 0.00 | 0.02 | 0.06 | 0.05 | 0.04 | 0.00 |
| 71.00 | 11.00 | CD | 0.08 | 0.00 | 0.20 | 1.51 | 0.00 | 0.08 | 0.00 | 0.01 | 0.01 | 0.00 |
| 111.00 | 7.00 | CD | 0.49 | 0.14 | 0.16 | 0.30 | 0.55 | 0.15 | 0.13 | 0.02 | 0.31 | 0.40 |
| 131.00 | 15.00 | CD | 0.20 | 0.00 | 0.14 | 1.03 | 0.37 | 0.00 | 0.00 | 0.00 | 0.00 | 0.00 |
| 163.00 | 17.50 | CD | 0.42 | 0.15 | 0.38 | 0.52 | 0.20 | 0.18 | 0.10 | 0.30 | 0.26 | 0.09 |
| 172.00 | 15.00 | CD | 0.39 | 0.09 | 0.04 | 1.04 | 0.19 | 0.45 | 0.10 | 0.01 | 0.91 | 0.22 |
| 180.00 | 15.00 | CD | 0.04 | 0.02 | 0.03 | 1.71 | 0.01 | 0.00 | 0.01 | 0.00 | 0.03 | 0.01 |
| 181.00 | 17.92 | CD | 0.02 | 0.03 | 0.03 | 1.72 | 0.02 | 0.02 | 0.03 | 0.02 | 0.10 | 0.03 |
| 188.00 | 11.00 | CD | 0.46 | 0.25 | 0.49 | 0.06 | 0.33 | 0.19 | 0.09 | 0.39 | 0.09 | 0.01 |
| 202.00 | 5.00 | CD | 0.16 | 0.00 | 0.02 | 1.55 | 0.11 | 0.00 | 0.00 | 0.00 | 0.00 | 0.00 |
| 1005.00 | 17.67 | CD | 0.06 | 0.06 | 0.09 | 1.50 | 0.12 | 0.08 | 0.05 | 0.13 | 0.35 | 0.06 |
| 1028.00 | 9.00 | CD | 0.23 | 0.05 | 0.06 | 0.92 | 0.42 | 0.32 | 0.06 | 0.06 | 1.20 | 0.57 |
| 1030.00 | 17.00 | CD | 0.19 | 0.03 | 0.05 | 1.21 | 0.28 | 0.27 | 0.05 | 0.06 | 1.01 | 0.49 |
| 6003.00 | 11.00 | CD | 0.23 | 0.81 | 0.08 | 0.19 | 0.23 | 0.32 | 0.93 | 0.11 | 0.20 | 0.30 |
| 6010.00 | 15.00 | CD | 1.02 | 0.01 | 0.00 | 0.47 | 0.18 | 0.50 | 0.02 | 0.00 | 0.54 | 0.01 |
| 6013.00 | 2.00 | CD | 0.51 | 0.22 | 0.22 | 0.13 | 0.50 | 0.08 | 0.02 | 0.06 | 0.12 | 0.14 |
| 6018.00 | 13.00 | CD | 0.34 | 0.04 | 0.01 | 0.92 | 0.20 | 0.33 | 0.01 | 0.01 | na | 0.03 |
| 6019.00 | 15.00 | CD | 0.66 | 0.08 | 0.11 | 0.48 | 0.28 | 0.02 | 0.01 | 0.13 | 0.14 | 0.04 |
| 6022.00 | 7.00 | CD | 1.20 | 0.00 | 0.00 | 0.38 | 0.10 | 0.06 | 0.00 | 0.00 | 0.05 | 0.09 |
| 6028.00 | 9.00 | CD | 0.76 | 0.06 | 0.00 | 0.29 | 0.55 | 0.00 | 0.00 | 0.00 | 0.00 | 0.00 |
| 6030.00 | 12.00 | CD | 0.93 | 0.06 | 0.01 | 0.19 | 0.44 | 0.47 | 0.01 | 0.01 | 0.04 | 0.43 |
| 6032.00 | 15.00 | CD | 0.60 | 0.06 | 0.04 | 0.83 | 0.16 | 0.72 | 0.01 | 0.05 | 0.99 | 0.16 |
| 6034.00 | 2.00 | CD | 0.98 | 0.06 | 0.01 | 0.16 | 0.40 | 0.08 | 0.03 | 0.02 | 0.03 | 0.06 |
| 6035.00 | 14.00 | CD | 0.01 | 0.01 | 0.01 | 1.76 | 0.00 | 0.02 | 0.02 | 0.02 | 0.01 | 0.00 |
| 6036.00 | 2.00 | CD | 0.67 | 0.07 | 0.10 | 0.71 | 0.15 | 0.63 | 0.04 | 0.07 | 0.90 | 0.11 |
| 20.00 | 17.92 | CON | 0.33 | 0.06 | 0.08 | 0.63 | 0.56 | 0.25 | 0.05 | 0.06 | 0.99 | 0.48 |
| 32.00 | 12.00 | CON | 0.80 | 0.08 | 0.09 | 0.30 | 0.32 | 0.15 | 0.04 | 0.06 | 0.43 | 0.12 |
| 35.00 | 16.00 | CON | 0.44 | 0.19 | 0.38 | 0.38 | 0.25 | 0.23 | 0.03 | 0.29 | 0.09 | 0.12 |
| 50.00 | 10.00 | CON | 0.05 | 0.02 | 0.03 | 1.70 | 0.01 | 0.02 | 0.01 | 0.00 | 0.03 | 0.02 |
| 54.00 | 17.67 | CON | 0.18 | 0.07 | 0.30 | 0.96 | 0.23 | 0.14 | 0.10 | 0.41 | 0.88 | 0.18 |
| 58.00 | 6.00 | CON | 0.56 | 0.14 | 0.12 | 0.35 | 0.43 | 0.14 | 0.08 | 0.03 | 0.43 | 0.29 |
| 77.00 | 17.92 | CON | 0.21 | 0.11 | 0.42 | 0.97 | 0.01 | 0.07 | 0.07 | 0.36 | 0.58 | 0.01 |
| 87.00 | 4.00 | CON | 0.34 | 0.04 | 0.01 | 0.92 | 0.20 | 0.33 | 0.01 | 0.01 | 0.00 | 0.03 |
| 88.00 | 4.00 | CON | 0.65 | 0.07 | 0.02 | 0.58 | 0.25 | 0.00 | 0.00 | 0.00 | 0.00 | 0.00 |
| 99.00 | 11.00 | CON | 0.67 | 0.09 | 0.20 | 0.38 | 0.31 | 0.00 | 0.00 | 0.00 | 0.00 | 0.00 |
| 130.00 | 6.00 | CON | 0.24 | 0.44 | 0.03 | 0.45 | 0.61 | 0.00 | 0.00 | 0.00 | 0.00 | 0.00 |
| 136.00 | 15.92 | CON | 0.30 | 0.05 | 0.01 | 1.18 | 0.25 | 0.07 | 0.04 | 0.02 | 0.16 | 0.08 |
| 158.00 | 15.00 | CON | 0.64 | 0.06 | 0.11 | 0.26 | 0.55 | 0.11 | 0.01 | 0.05 | 0.35 | 0.22 |
| 187.00 | 9.00 | CON | 0.07 | 0.00 | 0.10 | 1.19 | 0.38 | 0.00 | 0.00 | 0.00 | 0.00 | 0.00 |
| 191.00 | 12.00 | CON | 0.52 | 0.19 | 0.12 | 0.65 | 0.19 | 0.45 | 0.24 | 0.15 | 0.92 | 0.08 |
| 195.00 | 13.00 | CON | 0.45 | 0.26 | 0.22 | 0.62 | 0.11 | 0.47 | 0.23 | 0.06 | 0.88 | 0.06 |
| 196.00 | 15.00 | CON | 0.60 | 0.01 | 0.03 | 0.66 | 0.42 | 0.52 | 0.01 | 0.06 | 0.94 | 0.31 |
| 201.00 | 14.00 | CON | 0.36 | 0.10 | 0.12 | 0.70 | 0.42 | 0.32 | 0.15 | 0.10 | 0.98 | 0.52 |
| 205.00 | 6.00 | CON | 0.47 | 0.14 | 0.08 | 0.78 | 0.24 | 0.13 | 0.01 | 0.01 | 0.24 | 0.04 |
| 207.00 | 2.00 | CON | 0.33 | 0.01 | 0.13 | 0.99 | 0.27 | 0.18 | 0.02 | 0.02 | 0.07 | 0.14 |
| 1006.00 | 17.25 | CON | 0.01 | 0.04 | 0.02 | 1.76 | 0.01 | 0.01 | 0.03 | 0.00 | 0.01 | 0.01 |
| 5009.00 | 17.50 | CON | 0.21 | 0.13 | 0.09 | 1.19 | 0.13 | 0.33 | 0.16 | 0.11 | 0.92 | 0.19 |
| 6004.00 | 14.00 | CON | 0.74 | 0.11 | 0.10 | 0.16 | 0.48 | 0.27 | 0.05 | 0.06 | 0.16 | 0.26 |
| 6006.00 | 12.00 | CON | 0.08 | 0.00 | 0.74 | 0.23 | 0.65 | 0.12 | 0.00 | 0.86 | 0.32 | 0.92 |
| 6008.00 | 2.00 | CON | 0.44 | 0.95 | 0.12 | 0.03 | 0.02 | 0.44 | 0.68 | 0.17 | 0.04 | 0.03 |
| 6009.00 | 15.00 | CON | 0.39 | 0.95 | 0.07 | 0.08 | 0.08 | 0.42 | 0.73 | 0.05 | 0.11 | 0.15 |
| 6015.00 | 13.00 | CON | 0.39 | 0.10 | 0.16 | 0.48 | 0.53 | 0.05 | 0.02 | 0.12 | 0.39 | 0.28 |
| 6016.00 | 11.00 | CON | 0.23 | 0.81 | 0.08 | 0.19 | 0.23 | 0.32 | 0.93 | 0.11 | 0.20 | 0.30 |
| 6021.00 | 7.00 | CON | 0.02 | 0.28 | 0.00 | 0.52 | 0.37 | 0.02 | 0.31 | 0.00 | 0.00 | 0.52 |
| 79.00 | 16.00 | UC | 0.78 | 0.09 | 0.16 | 0.51 | 0.08 | 0.61 | 0.11 | 0.22 | 0.38 | 0.11 |
| 86.00 | 16.00 | UC | 0.27 | 0.00 | 0.15 | 0.00 | 1.23 | 0.00 | 0.00 | 0.00 | 0.00 | 0.00 |
| 119.00 | 8.00 | UC | 0.55 | 0.08 | 0.09 | 0.81 | 0.14 | 0.46 | 0.06 | 0.04 | 0.63 | 0.13 |
| 126.00 | 16.00 | UC | 0.44 | 0.08 | 0.09 | 0.64 | 0.43 | 0.25 | 0.08 | 0.08 | 0.80 | 0.33 |
| 192.00 | 14.00 | UC | 0.52 | 0.03 | 0.13 | 0.00 | 0.95 | 0.00 | 0.00 | 0.00 | 0.00 | 0.00 |
| 198.00 | 11.00 | UC | 0.67 | 0.51 | 0.27 | 0.00 | 0.06 | 0.06 | 0.01 | 0.01 | 0.00 | 0.03 |
| 1013.00 | 12.00 | UC | 0.30 | 0.18 | 0.13 | 0.89 | 0.19 | 0.42 | 0.18 | 0.12 | 1.07 | 0.21 |
| 6001.00 | 13.00 | UC | 0.22 | 0.11 | 0.68 | 0.70 | 0.02 | 0.05 | 0.06 | 0.04 | 0.07 | 0.00 |
| 6005.00 | 10.00 | UC | 0.44 | 0.05 | 0.01 | 0.75 | 0.22 | 0.29 | 0.02 | 0.01 | 0.24 | 0.04 |
| 6007.00 | 13.00 | UC | 0.54 | 0.08 | 0.22 | 0.21 | 0.58 | 0.00 | 0.00 | 0.00 | 0.00 | 0.00 |
| 6011.00 | 6.00 | UC | 0.99 | 0.06 | 0.01 | 0.17 | 0.38 | 0.35 | 0.01 | 0.01 | 0.04 | 0.32 |
| 6017.00 | 11.00 | UC | 0.67 | 0.07 | 0.10 | 0.71 | 0.15 | 0.63 | 0.04 | 0.07 | 0.90 | 0.11 |
| 6023.00 | 15.00 | UC | 0.15 | 0.01 | 0.50 | 1.09 | 0.00 | 0.17 | 0.02 | 0.41 | 0.61 | 0.00 |
| 6026.00 | 8.00 | UC | 0.64 | 0.07 | 0.42 | 0.28 | 0.23 | 0.39 | 0.09 | 0.34 | 0.40 | 0.32 |
| 6033.00 | 1.00 | UC | 0.53 | 0.16 | 0.52 | 0.21 | 0.23 | 0.38 | 0.11 | 0.54 | 0.18 | 0.18 |
| 6038.00 | 10.00 | UC | 0.17 | 0.03 | 0.09 | 1.51 | 0.00 | 0.01 | 0.04 | 0.01 | 0.06 | 0.00 |

**Suppl. Table 2. Classification of cloned sequences by RDP taxonomy**

| **Disease** | **Clone ID** | **Domain** | **Phylum** | **Class** | **Order** | **Family** | **Genus** |
| --- | --- | --- | --- | --- | --- | --- | --- |
| CD | 111.2 | Bacteria | Verrucomicrobia | Verrucomicrobiae | Verrucomicrobiales | Verrucomicrobiaceae | Akkermansia |
| CD | 111.16 | Bacteria | Bacteroidetes | Bacteroidia | Bacteroidales | Rikenellaceae | Alistipes |
| CD | 172.18 | Bacteria | Bacteroidetes | Bacteroidia | Bacteroidales | Rikenellaceae | Alistipes |
| CON | 5009.25 | Bacteria | Bacteroidetes | Bacteroidia | Bacteroidales | Rikenellaceae | Alistipes |
| CON | 5009.5 | Bacteria | Bacteroidetes | Bacteroidia | Bacteroidales | Rikenellaceae | Alistipes |
| CD | 111.23 | Bacteria | Bacteroidetes | Bacteroidia | Bacteroidales | Bacteroidaceae | Bacteroides |
| CD | 111.9 | Bacteria | Bacteroidetes | Bacteroidia | Bacteroidales | Bacteroidaceae | Bacteroides |
| CD | 172.12 | Bacteria | Bacteroidetes | Bacteroidia | Bacteroidales | Bacteroidaceae | Bacteroides |
| CD | 172.31 | Bacteria | Bacteroidetes | Bacteroidia | Bacteroidales | Bacteroidaceae | Bacteroides |
| CD | 6034.21 | Bacteria | Bacteroidetes | Bacteroidia | Bacteroidales | Bacteroidaceae | Bacteroides |
| CD | 6034.28 | Bacteria | Bacteroidetes | Bacteroidia | Bacteroidales | Bacteroidaceae | Bacteroides |
| CD | 6036.35 | Bacteria | Bacteroidetes | Bacteroidia | Bacteroidales | Bacteroidaceae | Bacteroides |
| CD | 6036.4 | Bacteria | Bacteroidetes | Bacteroidia | Bacteroidales | Bacteroidaceae | Bacteroides |
| CON | 32.5 | Bacteria | Bacteroidetes | Bacteroidia | Bacteroidales | Bacteroidaceae | Bacteroides |
| CON | 5009.17 | Bacteria | Bacteroidetes | Bacteroidia | Bacteroidales | Bacteroidaceae | Bacteroides |
| UC | 198.1 | Bacteria | Bacteroidetes | Bacteroidia | Bacteroidales | Bacteroidaceae | Bacteroides |
| UC | 198.26 | Bacteria | Bacteroidetes | Bacteroidia | Bacteroidales | Bacteroidaceae | Bacteroides |
| UC | 198.28 | Bacteria | Bacteroidetes | Bacteroidia | Bacteroidales | Bacteroidaceae | Bacteroides |
| UC | 198.36 | Bacteria | Bacteroidetes | Bacteroidia | Bacteroidales | Bacteroidaceae | Bacteroides |
| UC | 198.37 | Bacteria | Bacteroidetes | Bacteroidia | Bacteroidales | Bacteroidaceae | Bacteroides |
| UC | 198.39 | Bacteria | Bacteroidetes | Bacteroidia | Bacteroidales | Bacteroidaceae | Bacteroides |
| UC | 6005.28 | Bacteria | Bacteroidetes | Bacteroidia | Bacteroidales | Bacteroidaceae | Bacteroides |
| UC | 6005.8 | Bacteria | Bacteroidetes | Bacteroidia | Bacteroidales | Bacteroidaceae | Bacteroides |
| UC | 86.1 | Bacteria | Bacteroidetes | Bacteroidia | Bacteroidales | Bacteroidaceae | Bacteroides |
| UC | 86.12 | Bacteria | Bacteroidetes | Bacteroidia | Bacteroidales | Bacteroidaceae | Bacteroides |
| UC | 86.17 | Bacteria | Bacteroidetes | Bacteroidia | Bacteroidales | Bacteroidaceae | Bacteroides |
| UC | 86.2 | Bacteria | Bacteroidetes | Bacteroidia | Bacteroidales | Bacteroidaceae | Bacteroides |
| UC | 86.2 | Bacteria | Bacteroidetes | Bacteroidia | Bacteroidales | Bacteroidaceae | Bacteroides |
| UC | 86.26 | Bacteria | Bacteroidetes | Bacteroidia | Bacteroidales | Bacteroidaceae | Bacteroides |
| UC | 86.3 | Bacteria | Bacteroidetes | Bacteroidia | Bacteroidales | Bacteroidaceae | Bacteroides |
| UC | 86.32 | Bacteria | Bacteroidetes | Bacteroidia | Bacteroidales | Bacteroidaceae | Bacteroides |
| UC | 86.9 | Bacteria | Bacteroidetes | Bacteroidia | Bacteroidales | Bacteroidaceae | Bacteroides |
| UC | 87.1 | Bacteria | Bacteroidetes | Bacteroidia | Bacteroidales | Bacteroidaceae | Bacteroides |
| UC | 87.25 | Bacteria | Bacteroidetes | Bacteroidia | Bacteroidales | Bacteroidaceae | Bacteroides |
| UC | 87.28 | Bacteria | Bacteroidetes | Bacteroidia | Bacteroidales | Bacteroidaceae | Bacteroides |
| UC | 87.9 | Bacteria | Bacteroidetes | Bacteroidia | Bacteroidales | Bacteroidaceae | Bacteroides |
| CD | 6032.39 | Bacteria | Firmicutes | Clostridia | Clostridiales | Incertae Sedis XIV | Blautia |
| CD | 111.37 | Bacteria | Firmicutes | Clostridia | Clostridiales | Ruminococcaceae | Butyricicoccus |
| CD | 6036.21 | Bacteria | Firmicutes | Clostridia | Clostridiales | Clostridiaceae (Subfamily: Clostridiaceae 1) | Clostridium |
| CD | 6034.7 | Bacteria | Firmicutes | Clostridia | Clostridiales | Veillonellaceae | Dialister |
| CON | 5009.11 | Bacteria | Firmicutes | Clostridia | Clostridiales | Veillonellaceae | Dialister |
| CON | 5009.12 | Bacteria | Firmicutes | Clostridia | Clostridiales | Veillonellaceae | Dialister |
| CON | 5009.27 | Bacteria | Firmicutes | Clostridia | Clostridiales | Veillonellaceae | Dialister |
| CON | 5009.32 | Bacteria | Firmicutes | Clostridia | Clostridiales | Veillonellaceae | Dialister |
| CON | 5009.9 | Bacteria | Firmicutes | Clostridia | Clostridiales | Veillonellaceae | Dialister |
| UC | 6005.11 | Bacteria | Firmicutes | Clostridia | Clostridiales | Veillonellaceae | Dialister |
| UC | 6005.12 | Bacteria | Firmicutes | Clostridia | Clostridiales | Veillonellaceae | Dialister |
| UC | 6005.14 | Bacteria | Firmicutes | Clostridia | Clostridiales | Veillonellaceae | Dialister |
| UC | 6005.15 | Bacteria | Firmicutes | Clostridia | Clostridiales | Veillonellaceae | Dialister |
| UC | 6005.16 | Bacteria | Firmicutes | Clostridia | Clostridiales | Veillonellaceae | Dialister |
| UC | 6005.19 | Bacteria | Firmicutes | Clostridia | Clostridiales | Veillonellaceae | Dialister |
| UC | 6005.2 | Bacteria | Firmicutes | Clostridia | Clostridiales | Veillonellaceae | Dialister |
| UC | 6005.2 | Bacteria | Firmicutes | Clostridia | Clostridiales | Veillonellaceae | Dialister |
| UC | 6005.21 | Bacteria | Firmicutes | Clostridia | Clostridiales | Veillonellaceae | Dialister |
| UC | 6005.23 | Bacteria | Firmicutes | Clostridia | Clostridiales | Veillonellaceae | Dialister |
| UC | 6005.32 | Bacteria | Firmicutes | Clostridia | Clostridiales | Veillonellaceae | Dialister |
| UC | 6005.34 | Bacteria | Firmicutes | Clostridia | Clostridiales | Veillonellaceae | Dialister |
| UC | 6005.36 | Bacteria | Firmicutes | Clostridia | Clostridiales | Veillonellaceae | Dialister |
| UC | 6005.38 | Bacteria | Firmicutes | Clostridia | Clostridiales | Veillonellaceae | Dialister |
| UC | 6005.4 | Bacteria | Firmicutes | Clostridia | Clostridiales | Veillonellaceae | Dialister |
| CD | 172.1 | Bacteria | Firmicutes | Bacilli | Lactobacillales | Enterococcaceae | Enterococcus |
| CD | 172.21 | Bacteria | Firmicutes | Bacilli | Lactobacillales | Enterococcaceae | Enterococcus |
| CD | 172.23 | Bacteria | Firmicutes | Bacilli | Lactobacillales | Enterococcaceae | Enterococcus |
| CD | 172.25 | Bacteria | Firmicutes | Bacilli | Lactobacillales | Enterococcaceae | Enterococcus |
| CD | 172.26 | Bacteria | Firmicutes | Bacilli | Lactobacillales | Enterococcaceae | Enterococcus |
| CD | 172.27 | Bacteria | Firmicutes | Bacilli | Lactobacillales | Enterococcaceae | Enterococcus |
| CD | 172.29 | Bacteria | Firmicutes | Bacilli | Lactobacillales | Enterococcaceae | Enterococcus |
| CD | 172.3 | Bacteria | Firmicutes | Bacilli | Lactobacillales | Enterococcaceae | Enterococcus |
| CD | 172.33 | Bacteria | Firmicutes | Bacilli | Lactobacillales | Enterococcaceae | Enterococcus |
| CD | 172.34 | Bacteria | Firmicutes | Bacilli | Lactobacillales | Enterococcaceae | Enterococcus |
| CD | 172.35 | Bacteria | Firmicutes | Bacilli | Lactobacillales | Enterococcaceae | Enterococcus |
| CD | 172.38 | Bacteria | Firmicutes | Bacilli | Lactobacillales | Enterococcaceae | Enterococcus |
| CD | 172.39 | Bacteria | Firmicutes | Bacilli | Lactobacillales | Enterococcaceae | Enterococcus |
| CD | 172.6 | Bacteria | Firmicutes | Bacilli | Lactobacillales | Enterococcaceae | Enterococcus |
| CD | 1030.13 | Bacteria | Proteobacteria | Gammaproteobacteria | Enterobacteriales | Enterobacteriaceae | Escherichia/Shigella |
| CD | 1030.16 | Bacteria | Proteobacteria | Gammaproteobacteria | Enterobacteriales | Enterobacteriaceae | Escherichia/Shigella |
| CD | 1030.17 | Bacteria | Proteobacteria | Gammaproteobacteria | Enterobacteriales | Enterobacteriaceae | Escherichia/Shigella |
| CD | 1030.18 | Bacteria | Proteobacteria | Gammaproteobacteria | Enterobacteriales | Enterobacteriaceae | Escherichia/Shigella |
| CD | 1030.2 | Bacteria | Proteobacteria | Gammaproteobacteria | Enterobacteriales | Enterobacteriaceae | Escherichia/Shigella |
| CD | 1030.21 | Bacteria | Proteobacteria | Gammaproteobacteria | Enterobacteriales | Enterobacteriaceae | Escherichia/Shigella |
| CD | 1030.23 | Bacteria | Proteobacteria | Gammaproteobacteria | Enterobacteriales | Enterobacteriaceae | Escherichia/Shigella |
| CD | 1030.3 | Bacteria | Proteobacteria | Gammaproteobacteria | Enterobacteriales | Enterobacteriaceae | Escherichia/Shigella |
| CD | 1030.31 | Bacteria | Proteobacteria | Gammaproteobacteria | Enterobacteriales | Enterobacteriaceae | Escherichia/Shigella |
| CD | 1030.33 | Bacteria | Proteobacteria | Gammaproteobacteria | Enterobacteriales | Enterobacteriaceae | Escherichia/Shigella |
| CD | 1030.34 | Bacteria | Proteobacteria | Gammaproteobacteria | Enterobacteriales | Enterobacteriaceae | Escherichia/Shigella |
| CD | 1030.37 | Bacteria | Proteobacteria | Gammaproteobacteria | Enterobacteriales | Enterobacteriaceae | Escherichia/Shigella |
| CD | 1030.7 | Bacteria | Proteobacteria | Gammaproteobacteria | Enterobacteriales | Enterobacteriaceae | Escherichia/Shigella |
| CD | 1030.8 | Bacteria | Proteobacteria | Gammaproteobacteria | Enterobacteriales | Enterobacteriaceae | Escherichia/Shigella |
| CD | 172.13 | Bacteria | Proteobacteria | Gammaproteobacteria | Enterobacteriales | Enterobacteriaceae | Escherichia/Shigella |
| CD | 172.24 | Bacteria | Proteobacteria | Gammaproteobacteria | Enterobacteriales | Enterobacteriaceae | Escherichia/Shigella |
| CD | 6034.1 | Bacteria | Proteobacteria | Gammaproteobacteria | Enterobacteriales | Enterobacteriaceae | Escherichia/Shigella |
| CD | 6034.13 | Bacteria | Proteobacteria | Gammaproteobacteria | Enterobacteriales | Enterobacteriaceae | Escherichia/Shigella |
| CD | 6034.31 | Bacteria | Proteobacteria | Gammaproteobacteria | Enterobacteriales | Enterobacteriaceae | Escherichia/Shigella |
| CON | 32.11 | Bacteria | Proteobacteria | Gammaproteobacteria | Enterobacteriales | Enterobacteriaceae | Escherichia/Shigella |
| CON | 32.12 | Bacteria | Proteobacteria | Gammaproteobacteria | Enterobacteriales | Enterobacteriaceae | Escherichia/Shigella |
| CON | 32.15 | Bacteria | Proteobacteria | Gammaproteobacteria | Enterobacteriales | Enterobacteriaceae | Escherichia/Shigella |
| CON | 32.16 | Bacteria | Proteobacteria | Gammaproteobacteria | Enterobacteriales | Enterobacteriaceae | Escherichia/Shigella |
| CON | 32.19 | Bacteria | Proteobacteria | Gammaproteobacteria | Enterobacteriales | Enterobacteriaceae | Escherichia/Shigella |
| CON | 32.2 | Bacteria | Proteobacteria | Gammaproteobacteria | Enterobacteriales | Enterobacteriaceae | Escherichia/Shigella |
| CON | 32.21 | Bacteria | Proteobacteria | Gammaproteobacteria | Enterobacteriales | Enterobacteriaceae | Escherichia/Shigella |
| CON | 32.22 | Bacteria | Proteobacteria | Gammaproteobacteria | Enterobacteriales | Enterobacteriaceae | Escherichia/Shigella |
| CON | 32.23 | Bacteria | Proteobacteria | Gammaproteobacteria | Enterobacteriales | Enterobacteriaceae | Escherichia/Shigella |
| CON | 32.24 | Bacteria | Proteobacteria | Gammaproteobacteria | Enterobacteriales | Enterobacteriaceae | Escherichia/Shigella |
| CON | 32.25 | Bacteria | Proteobacteria | Gammaproteobacteria | Enterobacteriales | Enterobacteriaceae | Escherichia/Shigella |
| CON | 32.28 | Bacteria | Proteobacteria | Gammaproteobacteria | Enterobacteriales | Enterobacteriaceae | Escherichia/Shigella |
| CON | 32.3 | Bacteria | Proteobacteria | Gammaproteobacteria | Enterobacteriales | Enterobacteriaceae | Escherichia/Shigella |
| CON | 32.3 | Bacteria | Proteobacteria | Gammaproteobacteria | Enterobacteriales | Enterobacteriaceae | Escherichia/Shigella |
| CON | 32.35 | Bacteria | Proteobacteria | Gammaproteobacteria | Enterobacteriales | Enterobacteriaceae | Escherichia/Shigella |
| CON | 32.36 | Bacteria | Proteobacteria | Gammaproteobacteria | Enterobacteriales | Enterobacteriaceae | Escherichia/Shigella |
| CON | 32.37 | Bacteria | Proteobacteria | Gammaproteobacteria | Enterobacteriales | Enterobacteriaceae | Escherichia/Shigella |
| CON | 32.4 | Bacteria | Proteobacteria | Gammaproteobacteria | Enterobacteriales | Enterobacteriaceae | Escherichia/Shigella |
| CON | 32.6 | Bacteria | Proteobacteria | Gammaproteobacteria | Enterobacteriales | Enterobacteriaceae | Escherichia/Shigella |
| CON | 32.8 | Bacteria | Proteobacteria | Gammaproteobacteria | Enterobacteriales | Enterobacteriaceae | Escherichia/Shigella |
| UC | 119.1 | Bacteria | Proteobacteria | Gammaproteobacteria | Enterobacteriales | Enterobacteriaceae | Escherichia/Shigella |
| UC | 119.11 | Bacteria | Proteobacteria | Gammaproteobacteria | Enterobacteriales | Enterobacteriaceae | Escherichia/Shigella |
| UC | 119.12 | Bacteria | Proteobacteria | Gammaproteobacteria | Enterobacteriales | Enterobacteriaceae | Escherichia/Shigella |
| UC | 119.16 | Bacteria | Proteobacteria | Gammaproteobacteria | Enterobacteriales | Enterobacteriaceae | Escherichia/Shigella |
| UC | 119.17 | Bacteria | Proteobacteria | Gammaproteobacteria | Enterobacteriales | Enterobacteriaceae | Escherichia/Shigella |
| UC | 119.2 | Bacteria | Proteobacteria | Gammaproteobacteria | Enterobacteriales | Enterobacteriaceae | Escherichia/Shigella |
| UC | 119.21 | Bacteria | Proteobacteria | Gammaproteobacteria | Enterobacteriales | Enterobacteriaceae | Escherichia/Shigella |
| UC | 119.22 | Bacteria | Proteobacteria | Gammaproteobacteria | Enterobacteriales | Enterobacteriaceae | Escherichia/Shigella |
| UC | 119.23 | Bacteria | Proteobacteria | Gammaproteobacteria | Enterobacteriales | Enterobacteriaceae | Escherichia/Shigella |
| UC | 119.24 | Bacteria | Proteobacteria | Gammaproteobacteria | Enterobacteriales | Enterobacteriaceae | Escherichia/Shigella |
| UC | 119.25 | Bacteria | Proteobacteria | Gammaproteobacteria | Enterobacteriales | Enterobacteriaceae | Escherichia/Shigella |
| UC | 119.26 | Bacteria | Proteobacteria | Gammaproteobacteria | Enterobacteriales | Enterobacteriaceae | Escherichia/Shigella |
| UC | 119.29 | Bacteria | Proteobacteria | Gammaproteobacteria | Enterobacteriales | Enterobacteriaceae | Escherichia/Shigella |
| UC | 119.3 | Bacteria | Proteobacteria | Gammaproteobacteria | Enterobacteriales | Enterobacteriaceae | Escherichia/Shigella |
| UC | 119.31 | Bacteria | Proteobacteria | Gammaproteobacteria | Enterobacteriales | Enterobacteriaceae | Escherichia/Shigella |
| UC | 119.33 | Bacteria | Proteobacteria | Gammaproteobacteria | Enterobacteriales | Enterobacteriaceae | Escherichia/Shigella |
| UC | 119.35 | Bacteria | Proteobacteria | Gammaproteobacteria | Enterobacteriales | Enterobacteriaceae | Escherichia/Shigella |
| UC | 119.36 | Bacteria | Proteobacteria | Gammaproteobacteria | Enterobacteriales | Enterobacteriaceae | Escherichia/Shigella |
| UC | 119.39 | Bacteria | Proteobacteria | Gammaproteobacteria | Enterobacteriales | Enterobacteriaceae | Escherichia/Shigella |
| UC | 119.4 | Bacteria | Proteobacteria | Gammaproteobacteria | Enterobacteriales | Enterobacteriaceae | Escherichia/Shigella |
| UC | 119.5 | Bacteria | Proteobacteria | Gammaproteobacteria | Enterobacteriales | Enterobacteriaceae | Escherichia/Shigella |
| UC | 119.8 | Bacteria | Proteobacteria | Gammaproteobacteria | Enterobacteriales | Enterobacteriaceae | Escherichia/Shigella |
| CD | 6032.21 | Bacteria | Firmicutes | Clostridia | Clostridiales | Ruminococcaceae | Faecalibacterium |
| CON | 6009.19 | Bacteria | Firmicutes | Clostridia | Clostridiales | Ruminococcaceae | Faecalibacterium |
| CON | 6009.23 | Bacteria | Firmicutes | Clostridia | Clostridiales | Ruminococcaceae | Faecalibacterium |
| CON | 6009.9 | Bacteria | Firmicutes | Clostridia | Clostridiales | Ruminococcaceae | Faecalibacterium |
| UC | 86.14 | Bacteria | Firmicutes | Clostridia | Clostridiales | Ruminococcaceae | Faecalibacterium |
| UC | 86.19 | Bacteria | Firmicutes | Clostridia | Clostridiales | Ruminococcaceae | Faecalibacterium |
| UC | 86.21 | Bacteria | Firmicutes | Clostridia | Clostridiales | Ruminococcaceae | Faecalibacterium |
| UC | 86.25 | Bacteria | Firmicutes | Clostridia | Clostridiales | Ruminococcaceae | Faecalibacterium |
| UC | 86.29 | Bacteria | Firmicutes | Clostridia | Clostridiales | Ruminococcaceae | Faecalibacterium |
| UC | 86.3 | Bacteria | Firmicutes | Clostridia | Clostridiales | Ruminococcaceae | Faecalibacterium |
| UC | 86.38 | Bacteria | Firmicutes | Clostridia | Clostridiales | Ruminococcaceae | Faecalibacterium |
| UC | 86.5 | Bacteria | Firmicutes | Clostridia | Clostridiales | Ruminococcaceae | Faecalibacterium |
| CD | 6036.3 | Bacteria | Firmicutes | Bacilli | Lactobacillales | Carnobacteriaceae | Granulicatella |
| CD | 6036.5 | Bacteria | Firmicutes | Bacilli | Lactobacillales | Carnobacteriaceae | Granulicatella |
| CD | 6034.14 | Bacteria | Proteobacteria | Gammaproteobacteria | Pasteurellales | Pasteurellaceae | Haemophilus |
| CD | 6034.19 | Bacteria | Proteobacteria | Gammaproteobacteria | Pasteurellales | Pasteurellaceae | Haemophilus |
| CD | 6034.2 | Bacteria | Proteobacteria | Gammaproteobacteria | Pasteurellales | Pasteurellaceae | Haemophilus |
| CD | 6034.22 | Bacteria | Proteobacteria | Gammaproteobacteria | Pasteurellales | Pasteurellaceae | Haemophilus |
| CD | 6034.25 | Bacteria | Proteobacteria | Gammaproteobacteria | Pasteurellales | Pasteurellaceae | Haemophilus |
| CD | 6036.1 | Bacteria | Proteobacteria | Gammaproteobacteria | Pasteurellales | Pasteurellaceae | Haemophilus |
| CD | 6036.19 | Bacteria | Proteobacteria | Gammaproteobacteria | Pasteurellales | Pasteurellaceae | Haemophilus |
| CD | 6036.2 | Bacteria | Proteobacteria | Gammaproteobacteria | Pasteurellales | Pasteurellaceae | Haemophilus |
| CD | 6036.2 | Bacteria | Proteobacteria | Gammaproteobacteria | Pasteurellales | Pasteurellaceae | Haemophilus |
| CD | 6036.23 | Bacteria | Proteobacteria | Gammaproteobacteria | Pasteurellales | Pasteurellaceae | Haemophilus |
| CD | 6036.27 | Bacteria | Proteobacteria | Gammaproteobacteria | Pasteurellales | Pasteurellaceae | Haemophilus |
| CD | 6036.29 | Bacteria | Proteobacteria | Gammaproteobacteria | Pasteurellales | Pasteurellaceae | Haemophilus |
| CD | 6036.3 | Bacteria | Proteobacteria | Gammaproteobacteria | Pasteurellales | Pasteurellaceae | Haemophilus |
| CD | 6036.32 | Bacteria | Proteobacteria | Gammaproteobacteria | Pasteurellales | Pasteurellaceae | Haemophilus |
| CD | 6036.33 | Bacteria | Proteobacteria | Gammaproteobacteria | Pasteurellales | Pasteurellaceae | Haemophilus |
| CD | 6036.37 | Bacteria | Proteobacteria | Gammaproteobacteria | Pasteurellales | Pasteurellaceae | Haemophilus |
| UC | 198.1 | Bacteria | Proteobacteria | Gammaproteobacteria | Pasteurellales | Pasteurellaceae | Haemophilus |
| UC | 198.14 | Bacteria | Proteobacteria | Gammaproteobacteria | Pasteurellales | Pasteurellaceae | Haemophilus |
| UC | 198.16 | Bacteria | Proteobacteria | Gammaproteobacteria | Pasteurellales | Pasteurellaceae | Haemophilus |
| UC | 198.17 | Bacteria | Proteobacteria | Gammaproteobacteria | Pasteurellales | Pasteurellaceae | Haemophilus |
| UC | 198.2 | Bacteria | Proteobacteria | Gammaproteobacteria | Pasteurellales | Pasteurellaceae | Haemophilus |
| UC | 198.21 | Bacteria | Proteobacteria | Gammaproteobacteria | Pasteurellales | Pasteurellaceae | Haemophilus |
| UC | 198.22 | Bacteria | Proteobacteria | Gammaproteobacteria | Pasteurellales | Pasteurellaceae | Haemophilus |
| UC | 198.23 | Bacteria | Proteobacteria | Gammaproteobacteria | Pasteurellales | Pasteurellaceae | Haemophilus |
| UC | 198.24 | Bacteria | Proteobacteria | Gammaproteobacteria | Pasteurellales | Pasteurellaceae | Haemophilus |
| UC | 198.27 | Bacteria | Proteobacteria | Gammaproteobacteria | Pasteurellales | Pasteurellaceae | Haemophilus |
| UC | 198.32 | Bacteria | Proteobacteria | Gammaproteobacteria | Pasteurellales | Pasteurellaceae | Haemophilus |
| UC | 198.34 | Bacteria | Proteobacteria | Gammaproteobacteria | Pasteurellales | Pasteurellaceae | Haemophilus |
| UC | 198.35 | Bacteria | Proteobacteria | Gammaproteobacteria | Pasteurellales | Pasteurellaceae | Haemophilus |
| UC | 198.38 | Bacteria | Proteobacteria | Gammaproteobacteria | Pasteurellales | Pasteurellaceae | Haemophilus |
| UC | 198.4 | Bacteria | Proteobacteria | Gammaproteobacteria | Pasteurellales | Pasteurellaceae | Haemophilus |
| UC | 198.7 | Bacteria | Proteobacteria | Gammaproteobacteria | Pasteurellales | Pasteurellaceae | Haemophilus |
| UC | 198.8 | Bacteria | Proteobacteria | Gammaproteobacteria | Pasteurellales | Pasteurellaceae | Haemophilus |
| CD | 6032.31 | Bacteria | Firmicutes | Bacilli | Lactobacillales | Lactobacillaceae | Lactobacillus |
| UC | 6001.12 | Bacteria | Firmicutes | Bacilli | Lactobacillales | Lactobacillaceae | Lactobacillus |
| UC | 6001.15 | Bacteria | Firmicutes | Bacilli | Lactobacillales | Lactobacillaceae | Lactobacillus |
| UC | 6001.16 | Bacteria | Firmicutes | Bacilli | Lactobacillales | Lactobacillaceae | Lactobacillus |
| UC | 6001.2 | Bacteria | Firmicutes | Bacilli | Lactobacillales | Lactobacillaceae | Lactobacillus |
| UC | 6001.22 | Bacteria | Firmicutes | Bacilli | Lactobacillales | Lactobacillaceae | Lactobacillus |
| UC | 6001.24 | Bacteria | Firmicutes | Bacilli | Lactobacillales | Lactobacillaceae | Lactobacillus |
| UC | 6001.3 | Bacteria | Firmicutes | Bacilli | Lactobacillales | Lactobacillaceae | Lactobacillus |
| UC | 6001.3 | Bacteria | Firmicutes | Bacilli | Lactobacillales | Lactobacillaceae | Lactobacillus |
| UC | 6001.31 | Bacteria | Firmicutes | Bacilli | Lactobacillales | Lactobacillaceae | Lactobacillus |
| UC | 6001.33 | Bacteria | Firmicutes | Bacilli | Lactobacillales | Lactobacillaceae | Lactobacillus |
| UC | 6001.37 | Bacteria | Firmicutes | Bacilli | Lactobacillales | Lactobacillaceae | Lactobacillus |
| UC | 6001.38 | Bacteria | Firmicutes | Bacilli | Lactobacillales | Lactobacillaceae | Lactobacillus |
| UC | 6001.39 | Bacteria | Firmicutes | Bacilli | Lactobacillales | Lactobacillaceae | Lactobacillus |
| UC | 6001.4 | Bacteria | Firmicutes | Bacilli | Lactobacillales | Lactobacillaceae | Lactobacillus |
| UC | 6001.5 | Bacteria | Firmicutes | Bacilli | Lactobacillales | Lactobacillaceae | Lactobacillus |
| UC | 6001.6 | Bacteria | Firmicutes | Bacilli | Lactobacillales | Lactobacillaceae | Lactobacillus |
| UC | 6001.7 | Bacteria | Firmicutes | Bacilli | Lactobacillales | Lactobacillaceae | Lactobacillus |
| UC | 6001.11 | Bacteria | Firmicutes | Bacilli | Lactobacillales | Lactobacillaceae | Lactobacillus |
| CD | 6032.25 | Bacteria | Firmicutes | Bacilli | Lactobacillales | Streptococcaceae | Lactococcus |
| CD | 6032.6 | Bacteria | Firmicutes | Bacilli | Lactobacillales | Streptococcaceae | Lactococcus |
| CON | 5009.34 | Bacteria | Firmicutes | Clostridia | Clostridiales | Ruminococcaceae | Lactonifactor |
| CON | 32.33 | Bacteria | Firmicutes | Clostridia | Clostridiales | Veillonellaceae | Megamonas |
| UC | 198.6 | Bacteria | Proteobacteria | Betaproteobacteria | Neisseriales | Neisseriaceae | Neisseria |
| UC | 6005.24 | Bacteria | Bacteroidetes | Bacteroidia | Bacteroidales | Porphyromonadaceae | Odoribacter |
| CD | 172.2 | Bacteria | Firmicutes | Clostridia | Clostridiales | Ruminococcaceae | Oscillibacter |
| CON | 5009.29 | Bacteria | Firmicutes | Clostridia | Clostridiales | Ruminococcaceae | Oscillibacter |
| CON | 5009.33 | Bacteria | Firmicutes | Clostridia | Clostridiales | Ruminococcaceae | Oscillibacter |
| CD | 111.3 | Bacteria | Bacteroidetes | Bacteroidia | Bacteroidales | Porphyromonadaceae | Parabacteroides |
| CD | 111.32 | Bacteria | Bacteroidetes | Bacteroidia | Bacteroidales | Porphyromonadaceae | Parabacteroides |
| CON | 6009.6 | Bacteria | Bacteroidetes | Bacteroidia | Bacteroidales | Porphyromonadaceae | Parabacteroides |
| CON | 32.26 | Bacteria | Firmicutes | Clostridia | Clostridiales | Veillonellaceae | Phascolarctobacterium |
| UC | 6005.37 | Bacteria | Bacteroidetes | Bacteroidia | Bacteroidales | Prevotellaceae | Prevotella |
| UC | 6005.7 | Bacteria | Bacteroidetes | Bacteroidia | Bacteroidales | Prevotellaceae | Prevotella |
| CON | 6009.2 | Bacteria | Firmicutes | Clostridia | Clostridiales | Lachnospiraceae | Roseburia |
| CON | 6009.4 | Bacteria | Firmicutes | Clostridia | Clostridiales | Lachnospiraceae | Roseburia |
| CON | 6009.5 | Bacteria | Firmicutes | Clostridia | Clostridiales | Lachnospiraceae | Roseburia |
| CD | 6032.19 | Bacteria | Firmicutes | Clostridia | Clostridiales | Ruminococcaceae | Ruminococcus |
| CD | 6032.26 | Bacteria | Firmicutes | Clostridia | Clostridiales | Ruminococcaceae | Ruminococcus |
| CD | 6032.28 | Bacteria | Firmicutes | Clostridia | Clostridiales | Ruminococcaceae | Ruminococcus |
| CD | 6032.4 | Bacteria | Firmicutes | Bacilli | Lactobacillales | Streptococcaceae | Streptococcus |
| CD | 6036.24 | Bacteria | Firmicutes | Bacilli | Lactobacillales | Streptococcaceae | Streptococcus |
| CD | 6036.31 | Bacteria | Firmicutes | Bacilli | Lactobacillales | Streptococcaceae | Streptococcus |
| CD | 6036.6 | Bacteria | Firmicutes | Bacilli | Lactobacillales | Streptococcaceae | Streptococcus |
| CD | 6032.11 | Bacteria | Firmicutes | Clostridia | Clostridiales | Ruminococcaceae | Subdoligranulum |
| CD | 6032.12 | Bacteria | Firmicutes | Clostridia | Clostridiales | Ruminococcaceae | Subdoligranulum |
| CD | 6032.14 | Bacteria | Firmicutes | Clostridia | Clostridiales | Ruminococcaceae | Subdoligranulum |
| CD | 6032.17 | Bacteria | Firmicutes | Clostridia | Clostridiales | Ruminococcaceae | Subdoligranulum |
| CD | 6032.18 | Bacteria | Firmicutes | Clostridia | Clostridiales | Ruminococcaceae | Subdoligranulum |
| CD | 6032.22 | Bacteria | Firmicutes | Clostridia | Clostridiales | Ruminococcaceae | Subdoligranulum |
| CD | 6032.23 | Bacteria | Firmicutes | Clostridia | Clostridiales | Ruminococcaceae | Subdoligranulum |
| CD | 6032.27 | Bacteria | Firmicutes | Clostridia | Clostridiales | Ruminococcaceae | Subdoligranulum |
| CD | 6032.29 | Bacteria | Firmicutes | Clostridia | Clostridiales | Ruminococcaceae | Subdoligranulum |
| CD | 6032.3 | Bacteria | Firmicutes | Clostridia | Clostridiales | Ruminococcaceae | Subdoligranulum |
| CD | 6032.32 | Bacteria | Firmicutes | Clostridia | Clostridiales | Ruminococcaceae | Subdoligranulum |
| CD | 6032.35 | Bacteria | Firmicutes | Clostridia | Clostridiales | Ruminococcaceae | Subdoligranulum |
| CD | 6032.37 | Bacteria | Firmicutes | Clostridia | Clostridiales | Ruminococcaceae | Subdoligranulum |
| CD | 6032.7 | Bacteria | Firmicutes | Clostridia | Clostridiales | Ruminococcaceae | Subdoligranulum |
| CD | 6032.9 | Bacteria | Firmicutes | Clostridia | Clostridiales | Ruminococcaceae | Subdoligranulum |
| CD | 6032.15 | Bacteria | Firmicutes | Clostridia | Clostridiales | Ruminococcaceae | Subdoligranulum |
| CD | 6034.26 | Bacteria | Firmicutes | Clostridia | Clostridiales | Ruminococcaceae | Subdoligranulum |
| CON | 6009.1 | Bacteria | Firmicutes | Clostridia | Clostridiales | Ruminococcaceae | Subdoligranulum |
| CON | 6009.7 | Bacteria | Firmicutes | Clostridia | Clostridiales | Ruminococcaceae | Subdoligranulum |
| UC | 6005.17 | Bacteria | Firmicutes | Clostridia | Clostridiales | Ruminococcaceae | Subdoligranulum |
| UC | 6005.29 | Bacteria | Firmicutes | Clostridia | Clostridiales | Ruminococcaceae | Subdoligranulum |
| UC | 6005.35 | Bacteria | Firmicutes | Clostridia | Clostridiales | Ruminococcaceae | Subdoligranulum |
| CD | 172.19 | Bacteria | Proteobacteria | Betaproteobacteria | Burkholderiales | Alcaligenaceae | Sutterella |
| CD | 6036.1 | Bacteria | Firmicutes | Clostridia | Clostridiales | Peptostreptococcaceae | unclassified_"Peptostreptococcaceae |
| CD | 6032.13 | Bacteria | Firmicutes | Clostridia | Clostridiales | Lachnospiraceae | unclassified_Lachnospiraceae |
| CD | 6034.24 | Bacteria | Firmicutes | Clostridia | Clostridiales | Lachnospiraceae | unclassified_Lachnospiraceae |
| CON | 5009.14 | Bacteria | Firmicutes | Clostridia | Clostridiales | Lachnospiraceae | unclassified_Lachnospiraceae |
| CON | 5009.2 | Bacteria | Firmicutes | Clostridia | Clostridiales | Lachnospiraceae | unclassified_Lachnospiraceae |
| CD | 6034.3 | Bacteria | Proteobacteria | Gammaproteobacteria | Pasteurellales | Pasteurellaceae | unclassified_Pasteurellaceae |
| CD | 6034.6 | Bacteria | Proteobacteria | Gammaproteobacteria | Pasteurellales | Pasteurellaceae | unclassified_Pasteurellaceae |
| CD | 6036.13 | Bacteria | Proteobacteria | Gammaproteobacteria | Pasteurellales | Pasteurellaceae | unclassified_Pasteurellaceae |
| UC | 198.11 | Bacteria | Proteobacteria | Gammaproteobacteria | Pasteurellales | Pasteurellaceae | unclassified_Pasteurellaceae |
| UC | 198.12 | Bacteria | Proteobacteria | Gammaproteobacteria | Pasteurellales | Pasteurellaceae | unclassified_Pasteurellaceae |
| UC | 198.15 | Bacteria | Proteobacteria | Gammaproteobacteria | Pasteurellales | Pasteurellaceae | unclassified_Pasteurellaceae |
| UC | 198.3 | Bacteria | Proteobacteria | Gammaproteobacteria | Pasteurellales | Pasteurellaceae | unclassified_Pasteurellaceae |
| UC | 198.31 | Bacteria | Proteobacteria | Gammaproteobacteria | Pasteurellales | Pasteurellaceae | unclassified_Pasteurellaceae |
| UC | 198.33 | Bacteria | Proteobacteria | Gammaproteobacteria | Pasteurellales | Pasteurellaceae | unclassified_Pasteurellaceae |
| UC | 198.9 | Bacteria | Proteobacteria | Gammaproteobacteria | Pasteurellales | Pasteurellaceae | unclassified_Pasteurellaceae |
| CON | 6009.28 | Bacteria | Firmicutes | Clostridia | Clostridiales | Ruminococcaceae | unclassified_Ruminococcaceae |
| UC | 86.36 | Bacteria | Firmicutes | Clostridia | Clostridiales | Veillonellaceae | unclassified_Veillonellaceae |
| CD | 111.4 | Bacteria | Firmicutes | unclassified_"Firmicutes | |  |  |
| CD | 6032.15 | Bacteria | Firmicutes | Clostridia | Clostridiales | unclassified_Clostridiales | |
| CD | 6032.33 | Bacteria | Firmicutes | Clostridia | Clostridiales | unclassified_Clostridiales | |
| CD | 6032.34 | Bacteria | Firmicutes | Clostridia | Clostridiales | unclassified_Clostridiales | |
| CD | 6034.32 | Bacteria | Firmicutes | Clostridia | Clostridiales | unclassified_Clostridiales | |
| CD |  |  |  |  |  |  |  |
| CD | 6036.28 | Bacteria | Firmicutes | unclassified_Firmicutes | |  |  |
| CD | 6036.7 | Bacteria | TM7 | TM7_genera_incertae_sedis | | |  |
| CON | 32.1 | Bacteria | Bacteroidetes | Bacteroidia | Bacteroidales | unclassified_Bacteroidales | |
| CON | 5009.2 | Bacteria | Firmicutes | Clostridia | Clostridiales | unclassified_Clostridiales | |
| CON | 6009.22 | Bacteria | Firmicutes | Clostridia | Clostridiales | unclassified_Clostridiales | |
| UC | 6005.25 | Bacteria | Firmicutes | Clostridia | Clostridiales | unclassified_Clostridiales | |
| UC | 6005.3 | Bacteria | Firmicutes | Clostridia | Clostridiales | unclassified_Clostridiales | |
| UC | 86.24 | Bacteria | Firmicutes | Clostridia | Clostridiales | unclassified_Clostridiales | |
| UC | 86.28 | Bacteria | Firmicutes | Clostridia | Clostridiales | unclassified_Clostridiales | |
| UC | 86.39 | Bacteria | Firmicutes | Clostridia | Clostridiales | unclassified_Clostridiales | |
| UC | 86.4 | Bacteria | Firmicutes | Clostridia | Clostridiales | unclassified_Clostridiales | |
